# Supplementary material for: The interactome of intact mitochondria by cross-linking mass spectrometry provides evidence for coexisting respiratory supercomplexes
Source: Mol Cell Proteomics. 2017 Dec 8;17(2):216–32. doi: 10.1074/mcp.RA117.000470 (PMC5795388; doi:10.1074/mcp.RA117.000470)
Supplement: Supplemental Data [file supp_RA117.000470_133922_0_supp_23978_fzffwf.zip › spectra_annotation/mito_spectra_annotation/342-1-77-1-2-1.pdf]

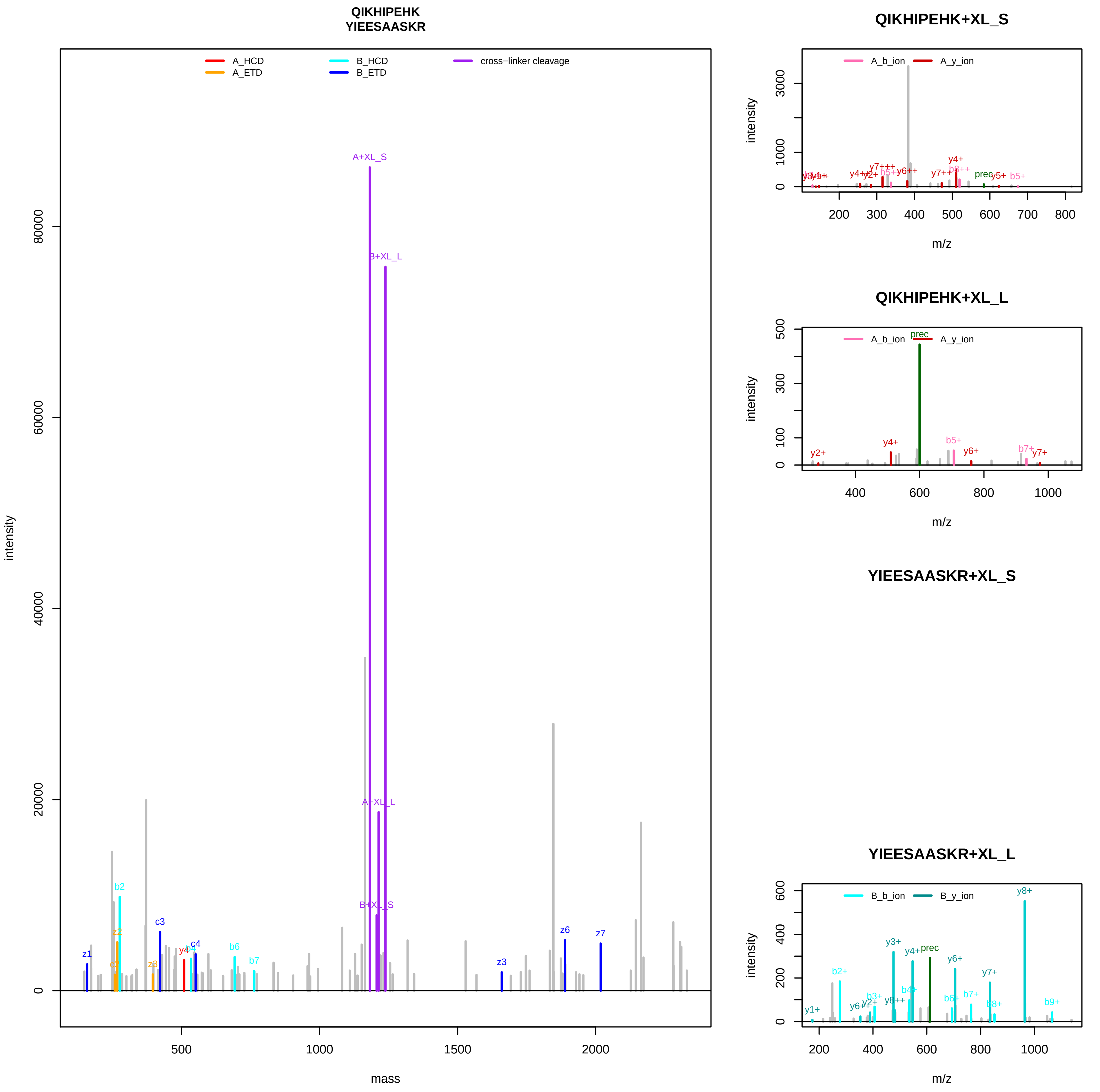
QIKHIPEHK
YIEESAASKR
A_HCD
A_ETD
B_HCD
B_ETD
cross−linker cleavage
A+XL_S
B+XL_L
A+XL_L
B+XL_S
intensity
mass
0
20000
40000
60000
80000
500
1000
1500
2000
z1
b2
c3
c4
b6
b7
z3
z6
z7
y4
QIKHIPEHK+XL_S
A_b_ion
A_y_ion
y4+
y7+++
y6++
y7++
prec
y5+
b5+
m/z
QIKHIPEHK+XL_L
y2+
y4+
b5+
y6+
b7+
y7+
YIEESAASKR+XL_S
YIEESAASKR+XL_L
B_b_ion
B_y_ion
y1+
b2+
y3+
y4+
y6+
b7+
y7+
y8+
b8+
b9+
